# Recent advances in enhanced polymer gels for profile control and water shutoff: A review

**DOI:** 10.3389/fchem.2023.1067094

**Published:** 2023-01-12

**Authors:** Siyu Lu, Qiwei Bo, Guang Zhao, Azizullah Shaikh, Caili Dai

**Affiliations:** ^1^ School of Petroleum Engineering, China University of Petroleum (East China), Qingdao, Shandong, China; ^2^ Sinopec International Petroleum Exploration and Production Corporation, Beijing, China; ^3^ Balochistan University of Information Technology, Engineering and Management Sciences Quetta, Balochistan, Pakistan

**Keywords:** enhanced polymer gels, crosslinking mechanism, enhanced mechanism, profile control, water shutoff

## Abstract

Polymer gels have been effectively employed as a water management material for profile control and water shutoff treatments in low-middle temperature and low-middle salinity reservoirs. However, most polymer gel systems have limitations under high temperature and salinity reservoir conditions, such as short gelation time, poor strength, and long-term instability. Therefore, several researchers have developed enhanced polymer gels to satisfy the water control requirements in high temperature and salinity reservoirs. This work reviews the five main types of enhanced polymer gels that have been developed so far: nano silica-enhanced gel systems, cellulose-enhanced gel systems, graphite-enhanced gel systems, oily sludge-enhanced gel systems, and foam-enhanced polymer gel systems. Further, this article investigates the fundamental properties, strengthening and crosslinking mechanisms, reservoir application conditions, and field applications of several enhanced polymer systems. In this paper, it is found that the addition of strengthening materials can increase the bound water content in the gel network and significantly improve the temperature and salt resistance of polymer gel, so as to cope with the application of profile control and water plugging in high temperature and high salt reservoirs. Moreover, it also offers references and future research directions for enhanced polymer gel systems.

## 1 Introduction

**FIGURE 1:**
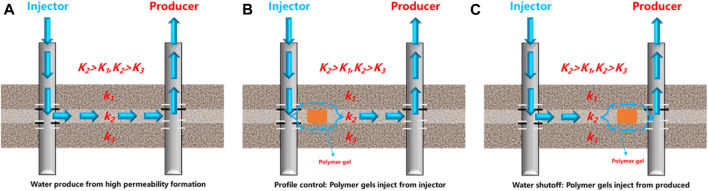
Schematic diagram of the polymer gel system applied to **(A)** water produce from high permeability formation, **(B)** profile control and **(C)** water shut-off treatments.

Presently, oilfields have developed various profile control and water shutoff techniques to address issues including excessive water production and serious reservoir heterogeneity (Majid et al., 2018). Polymer gel systems have become the most popular and promising water management material (Sydansk and Seright, 2007), due to the benefits of controllable gelation time, gel strength, and low cost, as shown in Figure 1. When the gelation solution is injected into the reservoir formation, the solution is converted to highly viscoelastic polymer gel. Thus the polymer gel systems can plug the high permeability layers, reduce the heterogeneity of the reservoir, and increase the sweep coefficient (Cheraghian and Hendraningrat, 2016). Thus, the residual oil can be easily driven out after water injection into the low and middle permeability layers. Generally, the conventional gel systems based on phenolic resin or chromium crosslinkers have been successfully applied to the reservoirs with temperatures (≤80°C) and salinities (≤50,000 mg/L) (Al-Muntasheri et al., 2010). The increasing application of high temperature and salinity reservoirs has emerged as a new hotspot with the continued development of conventional reservoirs (Ziegler, 2017). Yet, even after extensive water flooding, the high temperature and salinity reservoirs still face high produced water-cut. In these reservoirs, the profile control and water shutoff treatments are urgently being carried out (Zhao et al., 2006). However, conventional polymer gel systems have drawbacks of short gelation time, low strength, and long-term instability under high temperature and salinity reservoir conditions, leading to degraded gel systems, failure profile control, and water shutoff treatments (Bryant et al., 1997; Zhu et al., 2017).

Previous studies have compiled three reasons that make polymers unsuitable for application in high temperature and salinity reservoirs (Abdulbaki et al., 2014; Bai et al., 2015). Higher temperatures result in more rapid thermal motion of the molecules, which accelerates collisions between polymer and crosslinking agent molecules. This leads to the anomaly that the gelation time of the polymer gel systems is faster. However, the reduced gel formation time results in the polymer gel’s inability to penetrate the deep formation and a limited ability for water control. When the temperature rises, the chemical bonds between the polymer monomers are readily disrupted, resulting in polymer cracking. This makes the polymer gels weak and unable to plug the high permeability zones.

**FIGURE 2:**
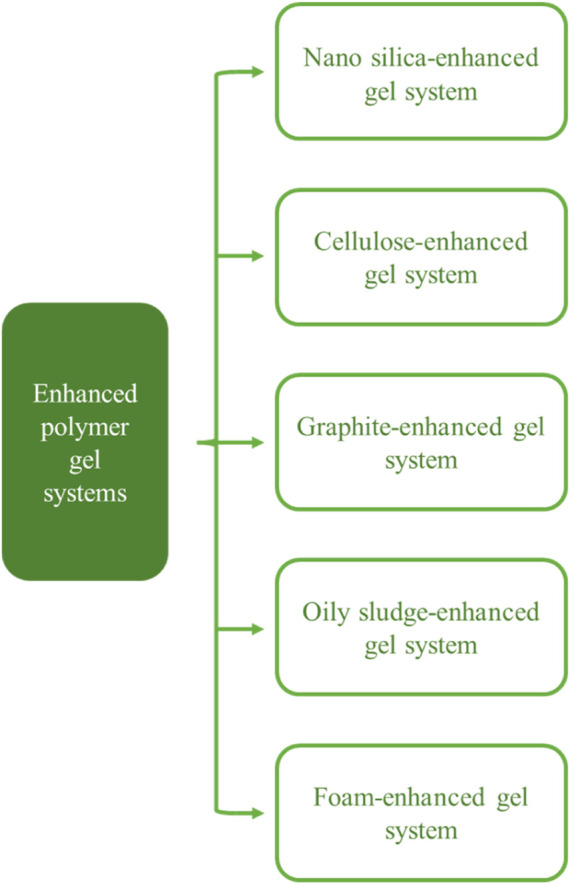
Classification of the enhanced polymer gel systems.

Prior studies have made excellent achievements in improving the temperature and salinity resistance of polymers and crosslinking agents in order to address the application issues of polymer gels in high temperature and salinity reservoirs in recent years. Additionally, to improve the temperature and salinity resistance of the polymer gels, some researchers have proposed adding some improved particles into the gel-forming fluid, such as nano-silica, cellulose, graphite, oily sludge, and foam; as shown in Figure 2. However, to the best of our knowledge, few papers have reviewed polymer-enhanced gel systems for water treatment in high-temperature and high-salt reservoirs, although more and more studies are being carried out. This study will evaluate an enhanced polymer gel system from the perspectives of chemistry and petroleum engineering in order to provide direction and discuss the potential for future applications in high temperature and salinity reservoirs.

## 2 Nano silica-enhanced gel system

Because of the gradual complexity of the reservoir conditions, conventional polymer gels like polyacrylamide (PAM) polymer gel systems and xanthan gum quickly break down or precipitate (Samuelson and Constien, 1996) when the reservoir temperature is more than 80°C and the salinity is more than 50,000 mg/L (Caulfield et al., 2002; Zhao et al., 2013). Moreover, the gelation time is shortened due to the limited crosslinking ability of the inorganic crosslinking agent, resulting in the instability of the gel system to migrate in the formation’s depth. Subsequently, nano silica particles are prepared and added to form nano silica-enhanced gel systems to increase strength and long-term stability, as shown in Table 1.

**TABLE 1:** Progress of nano silica-enhanced gel systems.

Nano silica-enhanced gel system				
Gelant formulation	Gelation temperature	Gelation time	Gel strength	References
Partially hydrolyzing polyacrylamide, Chromium acetate, Nano-silica	90°C	Not given	H-grade	Zareie et al. (2019)
Polyacrylamide, polyethyleneimine, Thiourea, Nano-silica	105°C	14 h	I-grade	Suleymanov and Shovgenov, (2021)
Partially hydrolyzing polyacrylamide, Chromium acetate, Nano-silica	Not given	9 h	H-grade	Fadil et al. (2020)
Nano-silica, Inorganic chloride	80°C	18 h	Not given	Huang et al. (2017)
Polyacrylamide, hydroquinone, hexamethylenetetramine, Nano-silica	Not given	16 h	Not given	Liu et al. (2017)

Zareie et al. (2019) prepared an enhanced gel system by partially hydrolyzed polyacrylamide (HPAM), chromium acetate crosslinking agent, and nano-silica particles. The gel strength reaches H-grade at 90°C with an elastic modulus of more than 26,000 Pa when 9% of nano-silica is added to the gelation solution. The gelation solution of this enhanced gel system has the characteristic of low viscosity at the initial gelation stage, which enables entering the in-depth formation. Also, the gel system could maintain a nano-silica network and its original characteristics without breaking when a strain of 100%–300% was applied, which justifies the high shearing stability of the fluid system.

**FIGURE 3:**
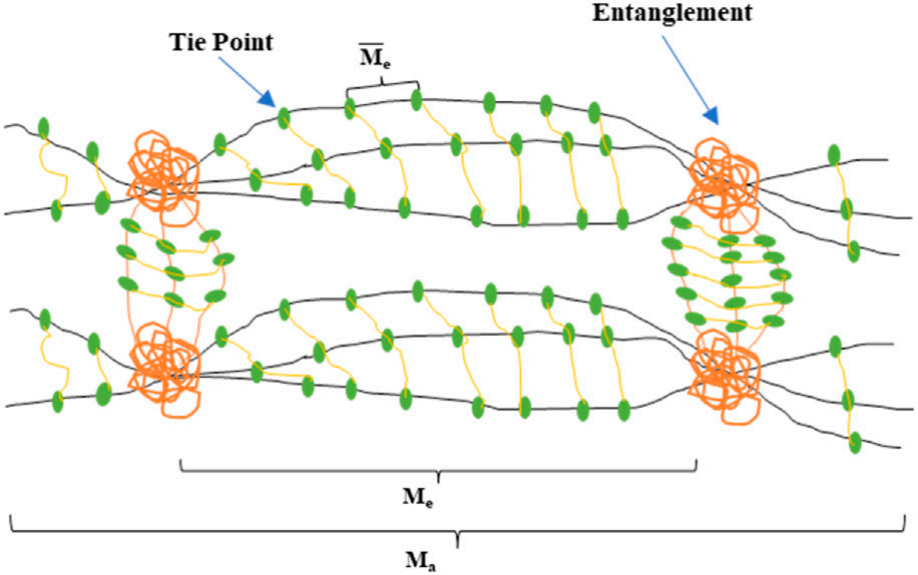
Nano silica-enhanced gel system network diagram (Zareie et al., 2019).

Moreover, the expansion rate of the system in distilled water can be increased rapidly within the first 30 min (Cassagnau, 2013). The gelation time increases due to the cage-like structure of nano-silica particles. These characteristics may bring good profile control and water shut-off effects. However, the study also found that the gelation time decreased when the concentration of nano silica was added to the gel by more than 12%. The reasons may be due to excessive crosslinking or agglomeration synergism of nano-silica in the gelation solution. The network structure and nano-silica particles act as a physical crosslinking agent and minimize the molecular weight between the two nodes in the network structure when compared to conventional HPAM gel systems. The molecular weight of this nano silica-enhanced gel system decreased from 726 g/mol to 257 g/mol, and the number of nodes increased by 55%, as shown in Figure 3. Accordingly, the nano silica-enhanced gel system significantly improves the temperature and salinity resistance.

**FIGURE 4:**
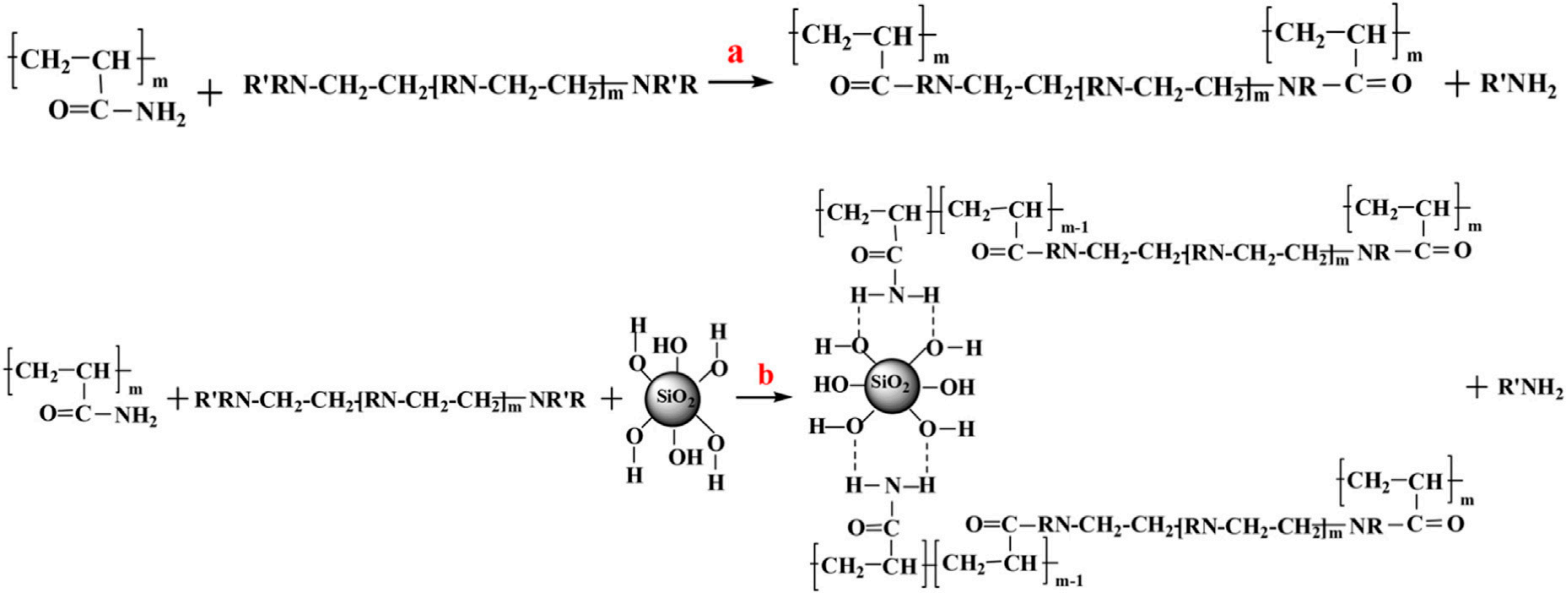
Schematic diagram of the enhanced gel system synthesis (Cassagnau, 2013).

Suleymanov and Shovgenov (2021) prepared an enhanced polymer gel system using PAM, polyethyleneimine (PEI), thiourea, and nano-silica at 211,688 mg/L salinity, as shown in Figure 4. When the nano-silica particles with an average particle size of 152.1 nm were added to the gelation solution, the gelation time and the gel strength at 105°C were 14 h, and I-grade, respectively. Generally, the lower bound water content and the easier dehydration of the gel system in high temperature and salinity reservoir conditions result in gel instability. The bound water content of the gel system increased by 19.532% after adding nano-silica particles. In addition, the system’s relative response factor (RRF) is still high after 30 days at 105°C through his research. As a result, the stability of enhanced gel systems brings a stronger plugging capability in porous media of formation. His paper summarizes the strengthening mechanisms of silica particles for two reasons. First, the hydrophilic nano-silica can act as a crosslinking agent, which generates silanol groups and increases the crosslink chance and density in the enhanced gel system. Second, the generated silanol groups interacting with the polymer free radicals through hydrogen bonds can significantly reduce the dehydration of the polymer gel system.

The studies also found that the system’s performance is degraded when the concentration of nano-silica particles exceeds 1%. The reason may be that the gel system’s strength is weakened by the over-crosslinking or self-aggregation of nano-silica particles. The concentration of nano-silica particles has a great influence on the stability of the gel. Therefore, while utilizing the nano-silica enhanced gel for profile control or water shutoff treatments, the concentration of nano-silica particles must be set at an appropriate value.

**FIGURE 5:**
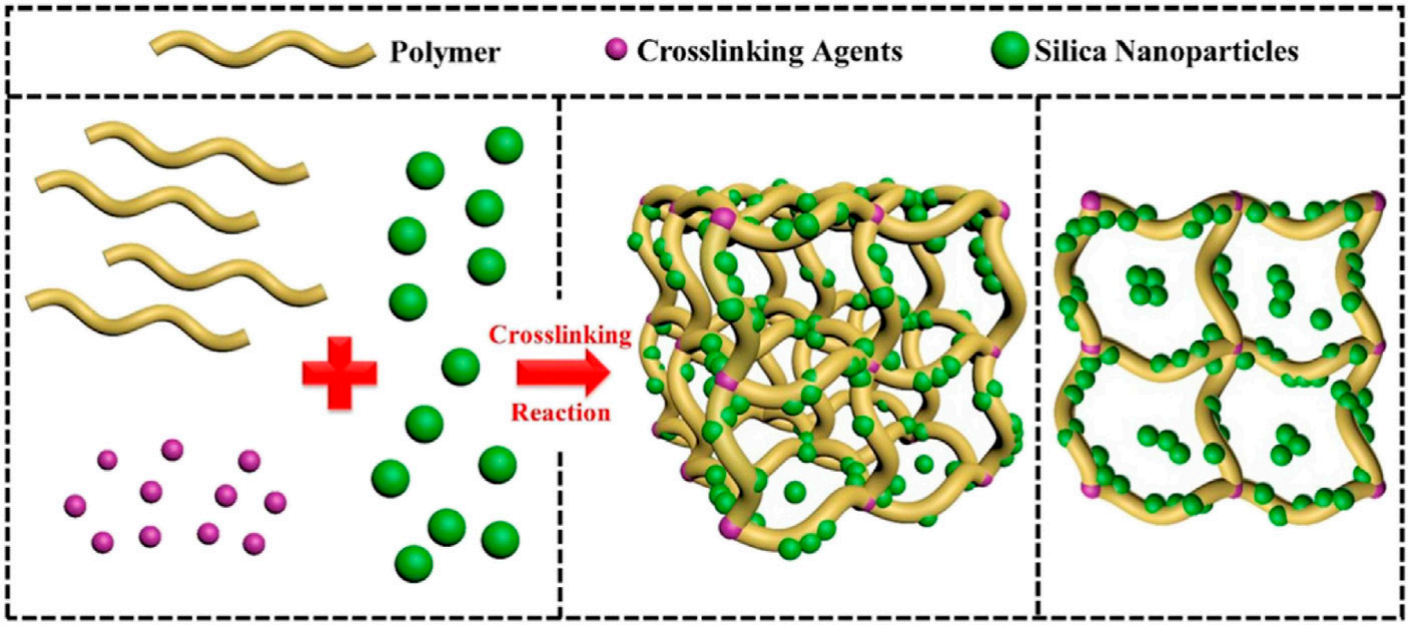
Strengthening mechanism of nano-silica particles to gel strength (Liu et al., 2017).

Liu et al. (2017) prepared an enhanced polymer gel system with polyacrylamide (PAM), hydroquinone (HQ), hexamethylenetetramine (HMTA), and nano-silica at high temperature and salinity conditions. The study found that adding nano-silica can significantly shorten the gelation time and improve the gel strength, elasticity, and viscosity of the enhanced gel system. Compared with the gel system without nano-silica, the maximum temperature tolerance of the enhanced gel system increased from 137.8°C to 155.5°C. When the nano-silica was added, the concentration of bound water in this nano silica-enhanced gel system increased from 22.5% to 39.9%. The nano silica-enhanced gel system forms a uniformly distributed three-dimensional network structure. The nano-silica significantly improves the network structure of this gel system to enhance the gel strength. Accordingly, the aggregation and arrangement of nano-silica particles can also be found in the polymer, chain bundles, and the network structure of the gel structure, as shown in Figure 5. In addition, the bound water content was significantly increased after adding nano-silica, which was beneficial to the hydrophilicity and thermal stability of the polymer gel.

**FIGURE 6:**
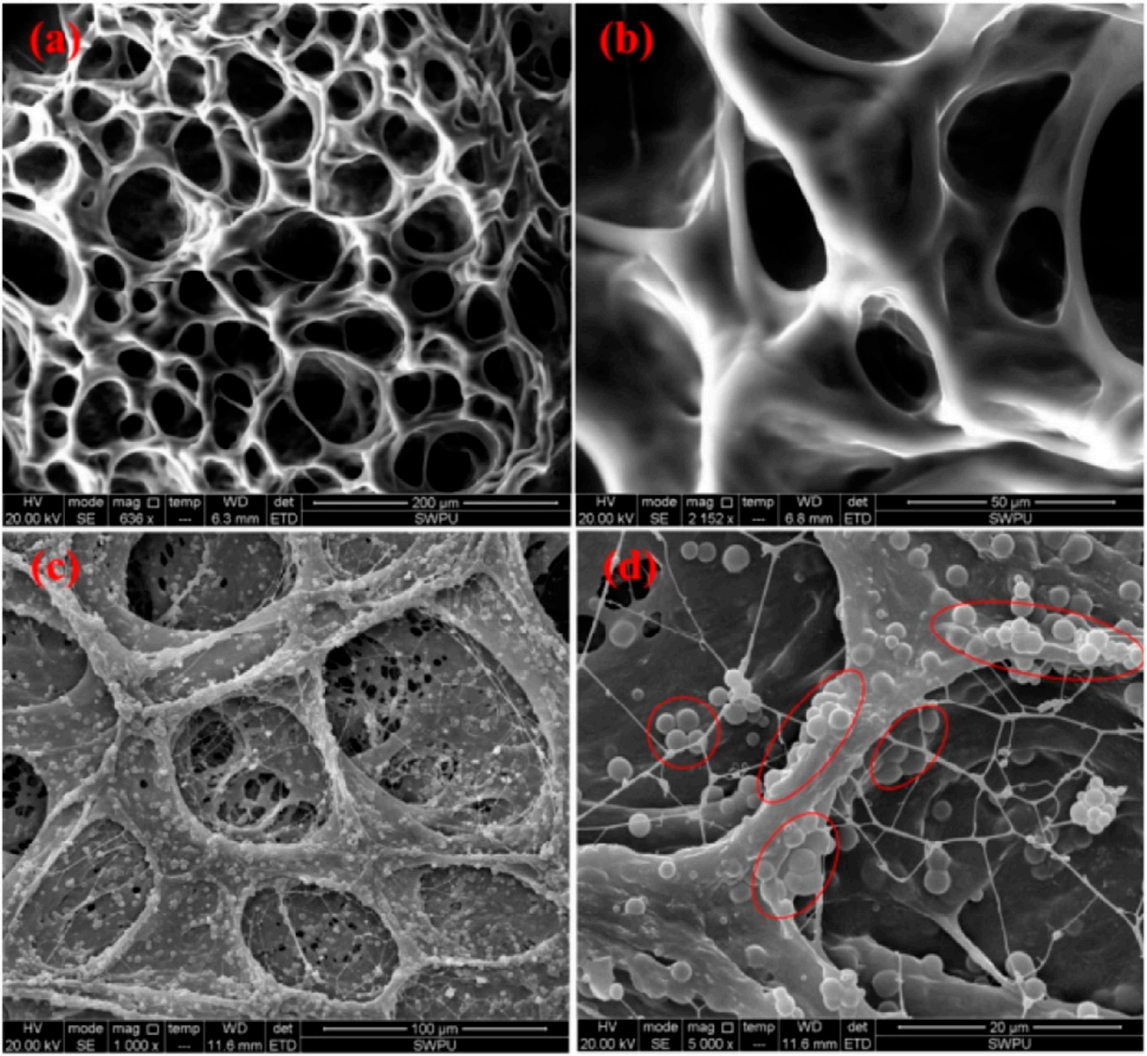
ESEM micrographs of gel samples prepared with different concentrations of nano-silica particles. **(A)**, **(B)** Without nano-silica particles. **(C)**, **(D)** .2 wt% (Liu et al., 2017).

Environmental scanning electron microscopy (ESEM) analysis revealed that the three-dimensional network structure was uniformly distributed throughout the gel system, as shown in Figure 6. The aggregation and arrangement of nano-silica particles can be found in gel’s polymer chain bundle and network structure. It helps to stabilize the gel system in high temperature and salinity reservoir conditions.

Fadil et al. (2020) used partially hydrolyzed polyacrylamide (HPAM), chromium acetate, and nano-silica particles to make an HPAM/Cr^3+^ nano silica-enhanced gel system. The study found that the gelation time was shortened with the increased concentration of nano-silica particles. When the concentration of the nano-silica was .3%, the gelation time and gel strength were respectively 9 h and H-grade, but the gel strength decreased from H-grade to G-grade after 25 h. However, the gelation time is shortened with the increase in nano-silica concentration. The short gelation time may not be conducive to transport in the formation’s porous media. The study found that the addition of nano-silica particles particularly increased the gel system’s strength and reduced its loss of strength. The reason may be that the presence of nano-silica particles in the polymer gel system promoted the crystal structure of the matrix so that the nano silica-enhanced gel system had good rigidity. Usually, the instability of the gel system is often related to its own thickening phenomenon (Pham and Hatzignatiou, 2016). Moreover, adding nano-silica particles to the gel system bring a denser gel network structure with a higher thickening effect, reducing free water and increasing bound water in the enhanced gel system, which also results in the enhancement of the stability.

Huang et al. (2017) constructed a nano silica-enhanced gel system at 150°C using nano-silica (particles ranging in size from 3 to 17 nm) and initiators. In this type of gel system, the higher concentration with the smaller nano-silica particles usually brings a faster-crosslinking speed. The study found that colloidal nanoparticles can remain stable in an alkaline solution, and the stability of the polymer gel system decreases with increasing temperature. The gelation time reached 18 h at 80°C, but the gelation time was affected by pH conditions. The lower the pH is, the shorter the gelation time. When the pH value was less than 7, the nano silica-enhanced gel system was unstable and easily aggregated. So, the gel solution must be prepared at an appropriate pH level when using this gel system for profile control and water shutoff treatments.

## 3 Cellulose-enhanced gel system

As a natural and renewable polymer material, cellulose has been widely used to prepare polymer gel systems (Chang et al., 2010). Also, some modified celluloses have high salinity and temperature resistance advantages (Dai et al., 2014). These materials provide the potential for preparing high temperature and salinity resistance gel systems. Compared with natural cellulose, cellulose nano-crystals (CNCs) are one-dimensional nano-materials (diameter 2–20 nm, length 50–300 nm), which not only have the renewable nature of natural cellulose but also have unique high crystallization (Hu et al., 2015), high surface ratio, low density, and excellent mechanical properties (Maiti et al., 2013). Different from conventional polymer materials, CNCs can be used as matrix enhancement to strengthen the crosslinking structure and can also be used as an interfacial adsorbent to improve the stability of the dispersion system. Therefore, adding CNCs can improve conventional polymer gel systems’ thermal stability and salinity resistance. Presently, many researchers have introduced CNCs into polymer gel systems to improve further thermal stability, salinity resistance, and mechanical strength (Goetz et al., 2009; Sampath et al., 2017). Adewole and Muritala (2019) previously summarized the application of several natural polymers in oilfields. From the application aspect of petroleum engineering in the future, this section highlights the fundamental performance, strengthening mechanism, and microstructure of cellulose-enhanced gel systems, as shown in Table 2.

**TABLE 2:** Progress of cellulose-enhanced gel systems.

Cellulose-enhanced gel systems				
Gelant formulation	Gelation temperature	Gelation time	Gel strength	References
C-PAM, phenolic resin crosslinker PF, ammonium chloride	80°C	Not given	Not given	Zhang et al. (2016)
TCNF, citric acid	Not given	Not given	Not given	Petroudy et al. (2018)

Zhang et al. (2016) prepared a cellulose-enhanced gel system using ultrafine cellulose, acrylamide graft copolymer, cationic polyacrylamide (C-PAM), phenolic resin crosslinker, and ammonium chloride. The viscosity of the cellulose-enhanced gel system can be up to 35,000 mPa s when gelled at 80°C and 100,000 mg/L. This gel system also has a good shear resistance. For example, the viscosity of this gelation solution holds over 60% of the initial viscosity when continuous shearing at 100 s^−1^. After gelation, the viscosity of the cellulose-enhanced gel system still remains at more than 80%. The high shear resistance of this gel system could keep a strong plugging capacity when transporting in porous media.

**FIGURE 7:**
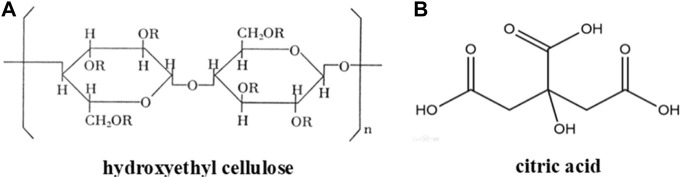
Chemical structures of **(A)** hydroxyethyl cellulose and **(B)** citric acid.

Petroudy et al. (2018) prepared a high water-absorption cellulose-enhanced gel system using sodium carboxymethyl cellulose and hydroxyethyl cellulose, also as well as tempo-oxidized cellulose nanofibers (TCNFs) and citric acid as crosslinking agents; as shown in Figure 7. The research showed that the gel system had a high swelling time, up to 200 g/g. The gel system had biodegradation and non-toxicity characteristics, while the ion sensitivity of the system decreased with increasing TCNF content. The strengthening mechanism is mainly based on multifunctional carboxylic acid that can react with cellulose. The multifunctional carboxylic acid is linked to the esterification reaction with the cellulose hydroxyl group, which further reacts with another cellulose hydroxyl group through the esterification reaction so that the cellulose plays a role in crosslinking (Zhou et al., 2013). Finally, this reaction resulted in the formation of a stable cellulose-enhanced gel system.

## 4 Graphite-enhanced gel system

Graphite particles have excellent characteristics, such as high-temperature tolerance, softness, self-lubrication, thermal stability, and easy modification (Du et al., 2018). These characteristics make it possible to prepare a graphite-enhanced gel system (Asghar et al., 2014). The high-temperature tolerance and high-salinity resistance were obviously improved when the graphite particles were embedded on the polymer branches or involved in the crosslinking reaction in the gel system (Yakovlev et al., 2006; Sengupta et al., 2011). In this paper, several of the basic properties of graphite-enhanced gel systems are summarized from the perspective of petroleum engineering, such as crosslinking mechanism, strengthening mechanism, and microscopic morphology, as shown in Table 3.

**TABLE 3:** Progress of graphite-enhanced gel systems.

Graphite-enhanced gel systems				
Gelant formulation	Gelation temperature	Gelation time	Gel strength	References
Polyacrylamide, hydroquinone, hexamethylenetetramine, graphene	150°C	Not given	Not given	Michael et al. (2020)
Polyacrylamide, hydroquinone, hexamethylenetetramine, graphene oxide	150°C	Not given	Not given	Michael et al. (2020)
NH-1, crosslinking agent I, crosslinking agent II, Graphite, CMC	160°C	Not given	Not given	Wang et al. (2020)
Polyacrylamide, nano-graphite, modified phenolic resin	130°C	Not given	Not given	Lv et al. (2020)

**FIGURE 8:**
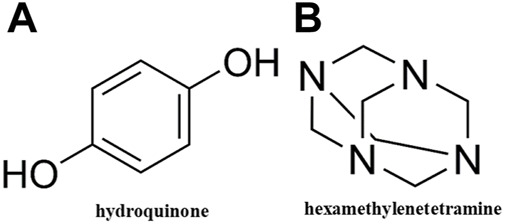
Chemical structures of **(A)** hydroquinone and **(B)** hexamethylenetetramine.

PAM-CG or PAM-GO graphite-enhanced gel systems were created by Michael et al. (2020) using polyacrylamide (PAM), hydroquinone (HQ), hexamethylenetetramine (HMTA), and graphite or graphite oxide (GO). The chemical structures of hydroquinone (HQ) and hexamethylenetetramine (HMTA) are shown in Figure 8. When .01% of CG particles are added, the elastic modulus and viscous modulus of the PAM-CG gel system reach 2,290 Pa and 415 Pa. Whereas, when .01% of GO particles are added, the elastic modulus and viscous modulus of the PAM-GO gel system reached 925 Pa and 158 Pa, respectively. The PAM-CG gel system has a stronger elastic and viscous modulus than the PAM-GO gel system. It may be related to the gel system’s various crosslinking modalities or crosslinking densities of graphene particles. Both these gel systems have excellent elasticity, and their water absorption capacity reached 27% and 25%. Furthermore, the degradation enthalpies of both gel systems reached 2153 J/g and 1350 J/g, respectively. This indicates that under high temperature and salinity reservoir conditions, both gel systems are long-term stable. The sand-pack flowing experiment showed that when both gel systems were formed in the porous media, the plugging rate could be above 80%. The study has proven that both graphite-enhanced gel systems had the advantages of good thermomechanical, water expansion, and self-healing properties.

**FIGURE 9:**
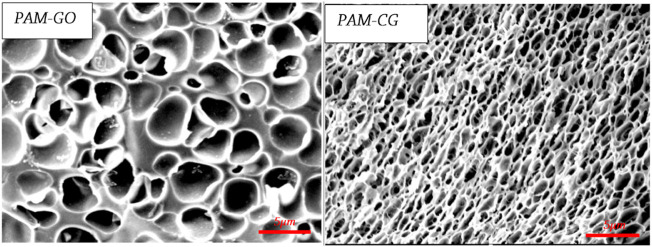
SEM images of PAM-CG and PAM-GO (Michael et al., 2020).

The scanning electron microscope (SEM) micrograph images demonstrate that both gel systems contain porous features, as shown in Figure 9. It can be observed that the PAM-CG gel system has a two-dimensional sheet-like structure with tiny hexagonal pores as a result of the presence of CG particles. However, the hexagonal pores were not observed in the microstructure of the PAM-GO gel system, which may be related to the hydrophilicity of GO particles.

Wang et al. (2020) prepared a graphite-enhanced gel system with polymer NH-1, crosslinking agents I and II, graphite particles, and suspension concentrate carboxymethyl cellulose (CMC); however, the precise crosslinker formula was not provided. The viscosity of the graphite-enhanced gel system reached 430,000 mPa s at 200°C. The graphite-enhanced gel system can be applied to reservoirs with temperatures from 160°C to 280°C and mineralization degrees from 8,000 mg/L to 10,000 mg/L. So, this gel system has great application potential for steam channel control in a heavy oil reservoir.

**FIGURE 10:**
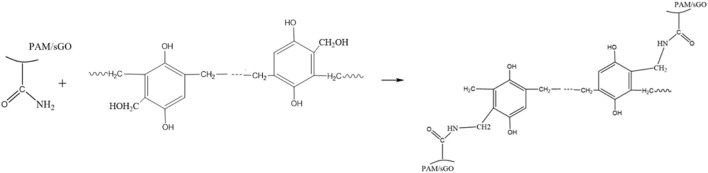
The gelation mechanism of the graphite-enhanced gel system (Lv et al., 2020).

Lv et al. (2020) prepared a high temperature and salinity resistant graphite-enhanced gel system with polyacrylamide (PAM), modified phenolic resin, and modified nano-graphite. This gel system can be crosslinked and have long-term stability under 130°C and 200,000 mg/L. This graphite-enhanced gel system performs well in terms of shear resistance and injection. Also, it has strong erosion resistance and plugging ability in different permeability formations. Additionally, the graphite-enhanced gel technology may avoid dehydration since it has more hydrophilicity than the conventional polymer system. The gelation mechanism is due to the crosslinking reaction between hydroxymethyl in phenolic resin and amide groups, forming a three-dimensional network gel; the crosslinking mechanism is shown in Figure 10.

The modified nano-graphite can improve the stability of the grid structure in the enhanced gel system and improve its water locking ability. The addition of modified nano graphite mainly strengthened the elastic modulus of the enhanced system, and the viscoelasticity of the enhanced gel system is more stable at high temperature. Compared with conventional PAM gel, the degradation rate of polymer in enhanced gel system is slower, the mass ratio of bound water is higher, and the gel is more hydrophilic. The modified nano-graphite mainly enhances the temperature resistance, salt resistance and shear resistance of the enhanced polymer through the characteristics of the inorganic particles, thereby significantly improving the stability of the enhanced gel system.

**FIGURE 11:**
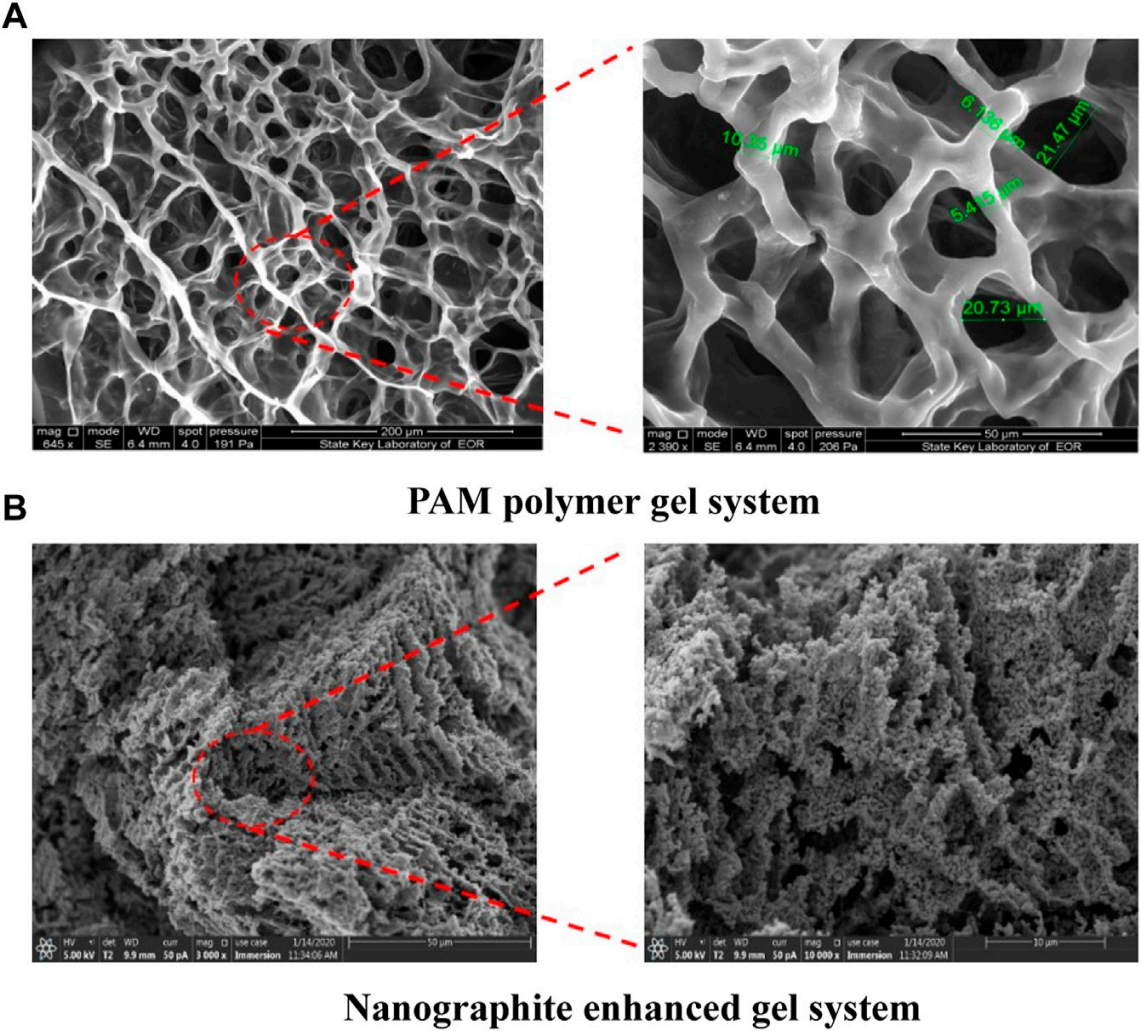
SEM images of the **(A)** PAM gel system and **(B)** nano-graphite enhanced gel system (Lv et al., 2020).

The SEM micrograph images of the PAM polymer gel system and nano-graphite enhanced gel system are shown in Figure 11. It can be observed that the enhanced gel system has a three-dimensional network structure of porous media with high crosslink density and close arrangement. In contrast, the network structures of the PAM gel system are relatively loose and have small pores. The special network structures of the enhanced gel system may bring high thermal stability under high temperature and salinity reservoir conditions.

## 5 Oily sludge-enhanced gel system

**FIGURE 12:**
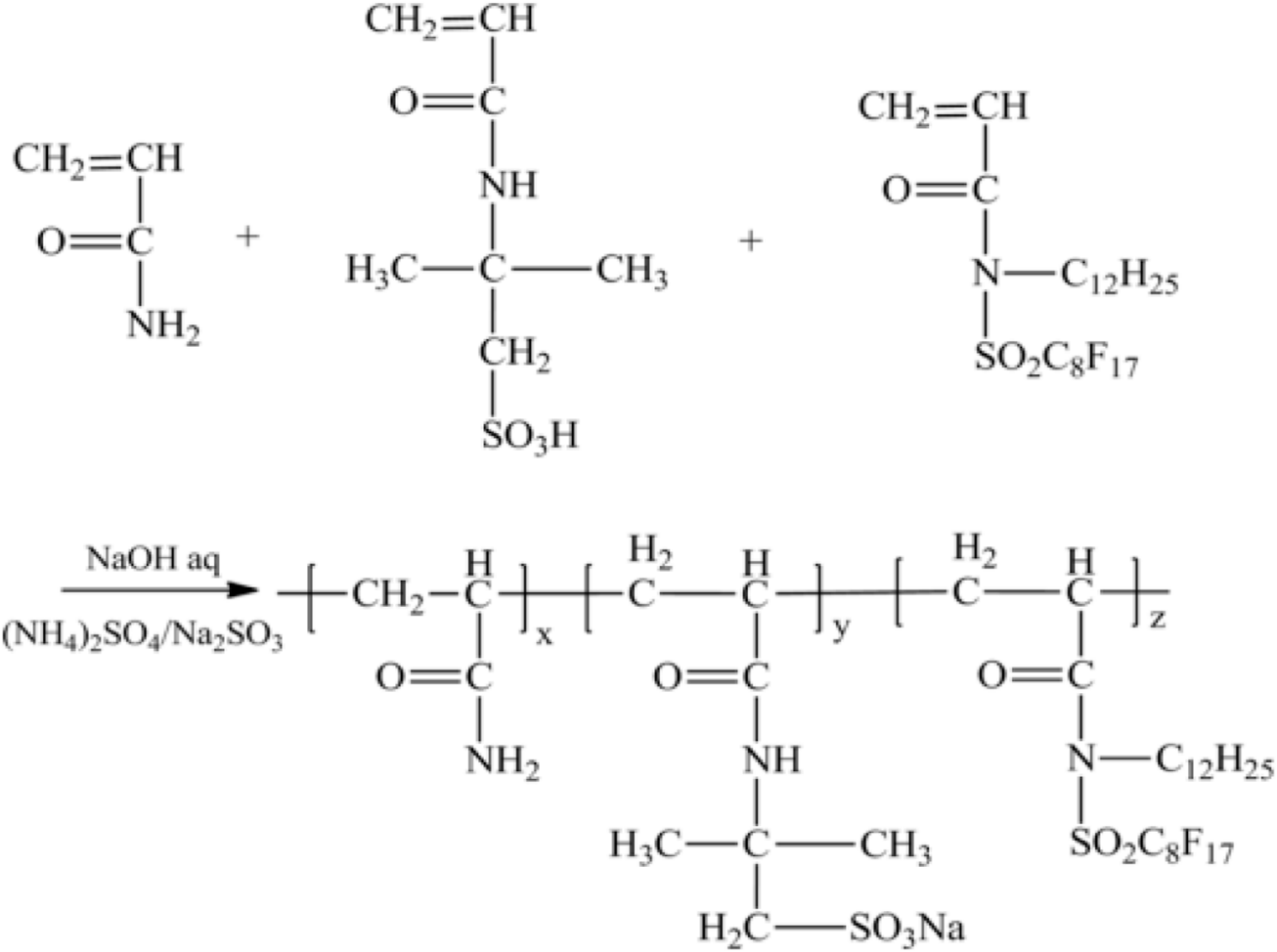
Synthesis route of FPAM (Chen et al., 2019).

Oily sludge is a solid waste produced in an oilfield development process and crude oil transportation and storage (Hu et al., 2013). If not managed appropriately, oily sludge will seriously impact the environment and human health (Caili et al., 2010). The use of oily sludge as a strengthening agent in gel systems has been studied with the development of profile control and water shutoff technologies, as shown in Table 4. Changqing Oilfield (Chen et al., 2019) produced an oily sludge-enhanced gel system using oily sludge, secondary alkyl sodium sulfonate (SAS-60), polyether (F-68), and fluorinated hydrophobically associating polyacrylamide (FPAM). The synthesis route of FPAM is shown in Figure 12. Based on this, a zirconium crosslinking agent was added to form an oily sludge-enhanced gel system. Due to the exit of amphiphilic hydrophobic association polymer, the suspended amount of oily sludge can be up to 40%. This gel system has been successfully used for profile control treatment in the oilfield. According to the production curve, the injection pressure rapidly increased by approximately 2.7 MPa during the injection period and then stabilized at 10.8 MPa. The water cut rate decreased by 11.5%, while the daily oil well production increased by .6 m^3^/d.

**TABLE 4:** Progress of oily sludge enhanced gel systems.

Oily sludge-enhanced gel systems				
Gelant formulation	Gelation Temperature	Gelation time	Gel strength	References
Secondary alkyl sodium sulfonate, polyether, zirconium crosslinking agent, oily sludge	Not given	Not given	2.7 MPa	Chen et al. (2019)
Polyacrylamide, oily sludge, suspending agent, crosslinking agent	80°C	22 h–28 h	Not given	Zhu et al. (2020)

Gudong Oilfield (Zhu et al., 2020) obtained an oily sludge enhanced gel system using polyacrylamide (PAM), suspending agent, oily sludge, and a crosslinking agent by adjusting the gelation time from 22 to 28 h. The gel system can maintain a high strength with a viscosity retention rate of more than 91% after aging for 180 days at 80°C. The sand-pack plugging experiment shows that the plugging rate and the breakthrough pressure can reach 94% and 6.2 MPa, respectively. The experimental data indicate that the enhanced gel system has strong anti-aging effectiveness, better cost management, and potential application prospects.

## 6 Foam-enhanced gel system

The conventional and enhanced gel systems have been successfully used for profile control and water shutoff treatments (Kabir, 2001). Due to the gel system’s capability to efficiently plug high permeability zones, the injected water will turn to intermediate and low permeability zones, followed by the increased swept volume (Sengupta et al., 2012). However, these gel systems cannot reduce the oil-water interfacial tension. While the foam systems have selectively plugging capability and oil-water interfacial tension reduction capacity (Miller and Fogler, 1995). However, the conventional foam is instability when flowing in porous media of reservoir formation. Considering the advantages and disadvantages of gel and foam systems, previous researchers have done many works on the progress of foam-enhanced gel systems (Wassmuth et al., 2000). The foam-enhanced gel systems are usually composed of polymer, crosslinker, foaming agent, and gas (Sydansk, 1994; Coskuner et al., 2015). When foam-enhanced gel systems form in the reservoir formation (Liu et al., 2006), the gel systems reduce the oil-water interfacial tension (Zhong et al., 2017), while the foam systems considerably increase the stability (Zhu et al., 2014; Chaudhary et al., 2021). Compared with conventional gel systems, foam-enhanced gels have higher temperature and salt resistance stability. Because the foam-enhanced gels have a long half-life and a large plugging radius, so the foam-enhanced gels can long-term stable plugging high permeability layer, so as to achieve more effective profile control and water plugging, as shown in Table 5.

**TABLE 5:** Progress of foam-enhanced gel systems.

Foam-enhanced gel systems				
Gelant formulation	Gelation Temperature	Gelation time	Gel strength	References
Comb polymer, phenolic resin, N_2_	100°C	14 h	Not given	Zhao et al. (2015)
Partially hydrolyzing polyacrylamide, Cr^3+^, sodium sulfite, N_2_	Not given	Not given	Not given	Qing et al. (2009)
Polyacrylonitrile, phenolic resin, alkali lignin	175°C	3.5 h	G-grade	Li et al. (2022)

**FIGURE 13:**
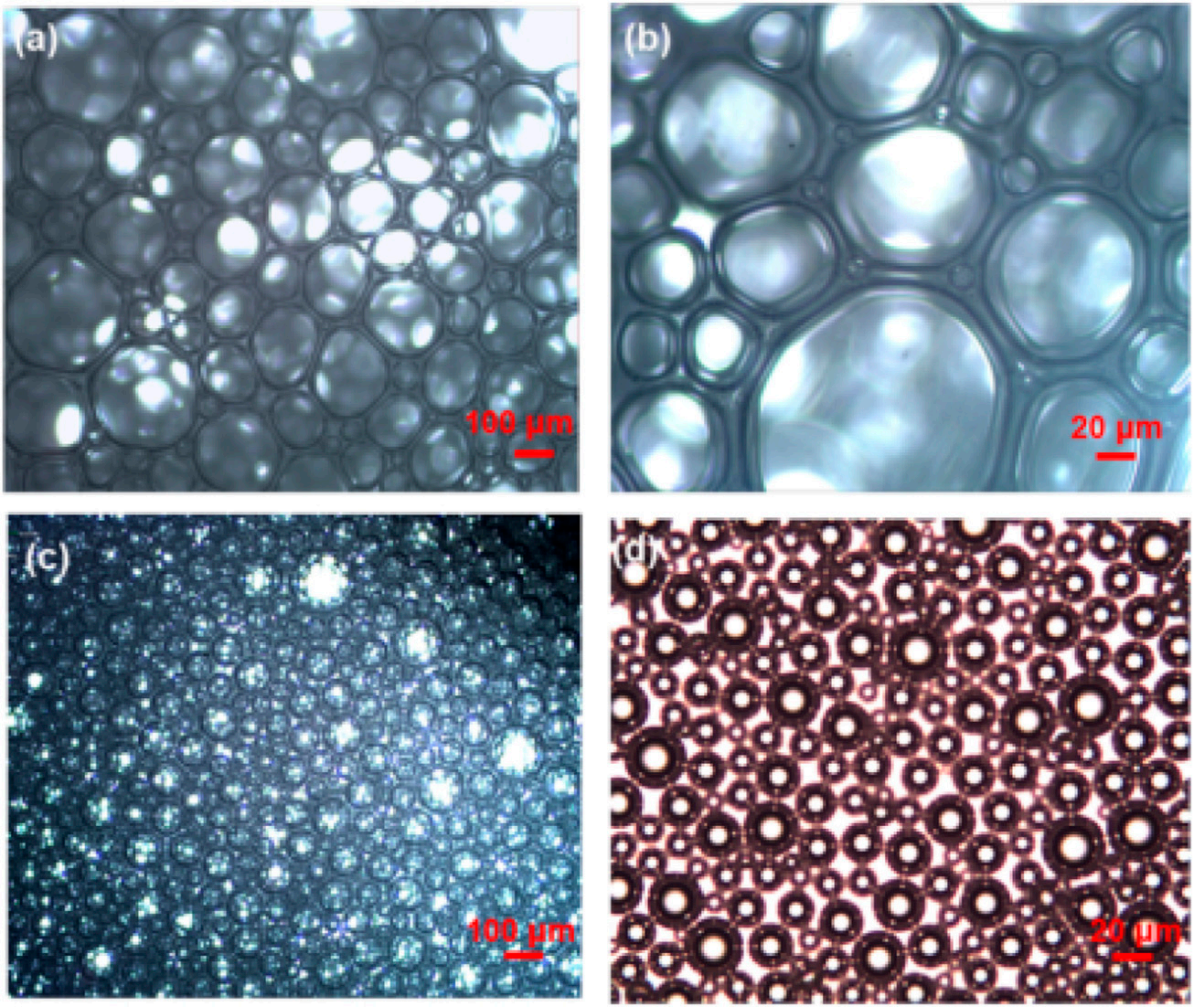
Morphology of conventional aqueous foam and foam-enhanced gel. **(A)** and **(B)** Conventional aqueous foam with only .3% surfactant; **(C)** and **(D)** foam gel (.3% surfactant + .3% comb polymer + .6% phenolic resin cross-linker) (Zhao et al., 2015).

Zhao et al. (2015) successfully prepared a long-term stable foam-enhanced gel system with comb polymer, phenolic resin crosslinker, betaine surfactant, and nitrogen gas. The system can be crosslinked at 100°C with the gelation time adjusting from 5 to 30 h. The foam-enhanced gel system has a longer half-life than the conventional aqueous foam. The half-life and foaming volume of the foam-enhanced gel system is increased by 10 times and 1.5 times, respectively. In addition, the half-life and volume of the foam-enhanced gel system remain almost unchanged after 28 h of gel formation. The viscosity of the foam-enhanced gel system was significantly increased due to the crosslinking reaction. While the interfacial tension was also reduced, resulting in a decrease in the foam volume. The foam-enhanced gel system has a denser and more consistent structure when compared to the conventional foam system, as shown in Figure 13. However, due to the increase in viscosity, the thickness and strength of the foam film are also increased, which enhances the stability and plugging ability of the foam in the porous medium.

For profile control treatments, the foam-enhanced gel technology has been successfully applied in 6 water injection wells in the Henan Oilfield. The injection pressure and oil production from these wells showed a sharp decline up to December 2011. The well-test data shows that the formation heterogeneity is very serious, and the reservoir temperature is as high as 100°C. After the foam-enhanced gel system treatments, an increase in five parameters (injection pressure, water injection, pressure index, full degree, and injectivity index) (Zhao et al., 2006; Zhao, 2011) was observed, which proves that the foam-enhanced gel system has a good in-depth profile control ability.

**FIGURE 14:**
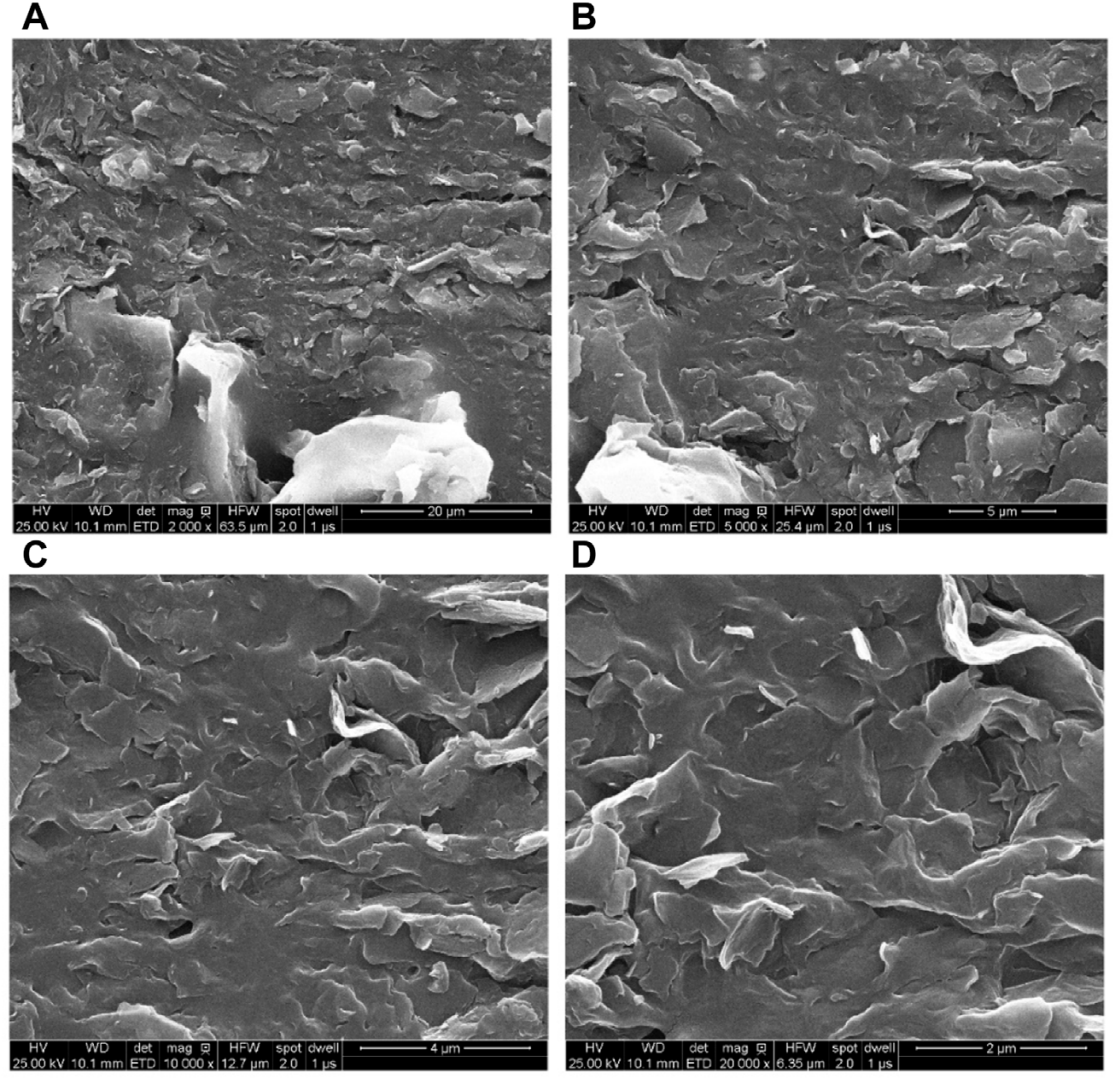
SEM image of the mature gel. The magnification of **(A)**, **(B**), **(C)** and **(D)** are 2000, 5,000, 10,000, and 20,000 respectively (Li et al., 2022).

Qing et al. (2009) prepared a foam-enhanced gel system with HPAM, sodium dichromate, sodium sulfite, and nitrogen. The foam-enhanced gel system was successfully applied to the Well H1304 of the Huoshaoshan fractured oilfield. After injection of 9,413 m^3^ foam-enhanced gel system, the oil production increased by 7,800 m^3^, and the input-output ratio was around 1:3. Li et al. (2022) prepared a high temperature and salinity resistance foam-enhanced gel system with polyacrylonitrile, phenolic resin, alkali lignin, foaming agent, and nitrogen. This foam-enhanced gel system is useful in reservoirs with high-temperature (175°C) and high-salinity (200,000 mg/L). Through the sand-pack flowing experiments, the foam-enhanced gel system has a stronger plugging capacity than the conventional foam gel systems. From SEM images (Figure 14), a dense and compact network structure may be produced when the foam-enhanced gel system is developed. The structure improves the liquid foam film’s viscoelasticity, which slows the escape of gas from inside the foam and promotes foam stability.

## 7 Conclusion

This paper reviews the progress of several enhanced polymer gel systems for profile control and water shutoff treatments. The most recent advancements in the present enhanced polymer gel systems comprise nano silica-enhanced gel systems, cellulose-enhanced gel systems, graphite-enhanced gel systems, oily sludge-enhanced gel systems, and foam-enhanced gel systems.

According to the present research level and the existing reservoir development scenario, an enhanced polymer gel system is an essential development direction for high water production control in high temperature and salinity reservoirs. Nano silica, graphite, cellulose, oily sludge and other strengthening materials can be used to strengthen the gel to deal with high temperature and high salt reservoir conditions. Nano-silica can reduce the bound water content and participate in the construction of the three-dimensional structure of the gel system, and can also improve the hydrophilicity of the gel systems. The addition of nano-cellulose enables the gel to crosslink between the cellulose chains to increase the strength. Due to its good plasticity and its lamellar structure, nano-graphite can also improve the temperature and salt resistance of the gel systems. Previous researchers have made many works on the enhanced gel system’s composition, crosslink and enhanced mechanisms, on-site applications, and so on. For prospective researchers, these findings serve as an excellent reference and source of development ideas. It has the enormous potential to produce more perceptive research findings for enhanced polymer gel systems.
